# Supplementary figures and images for: Quantifying participant distress: Validity and applicability of a distress measure to evaluate harm in quantitative assessments
Source: PLoS One. 2025 Jul 2;20(7):e0326957. doi: 10.1371/journal.pone.0326957 (PMC12220986; doi:10.1371/journal.pone.0326957)

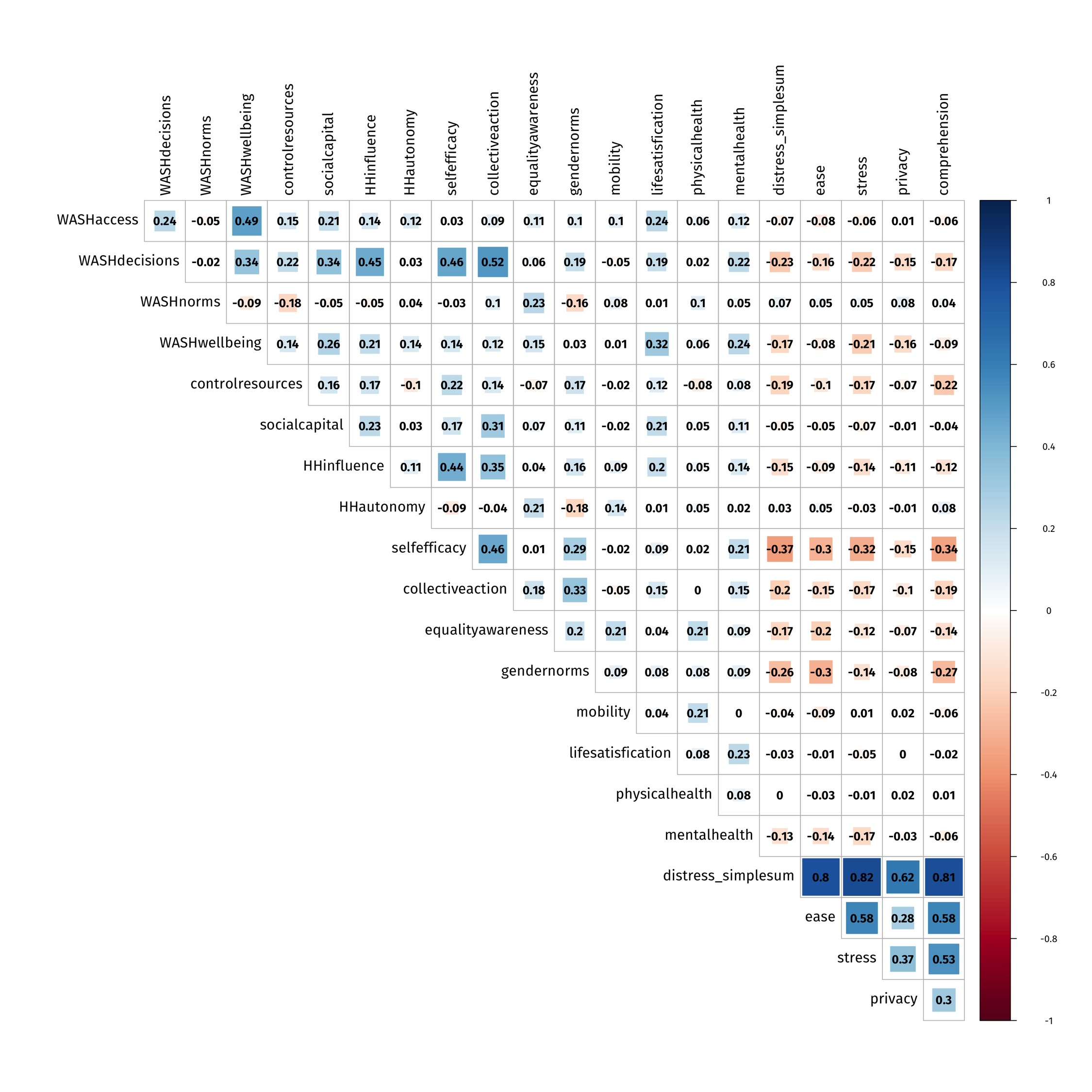

Supplement: S1 Fig — (TIF) [file pone.0326957.s003.tif]

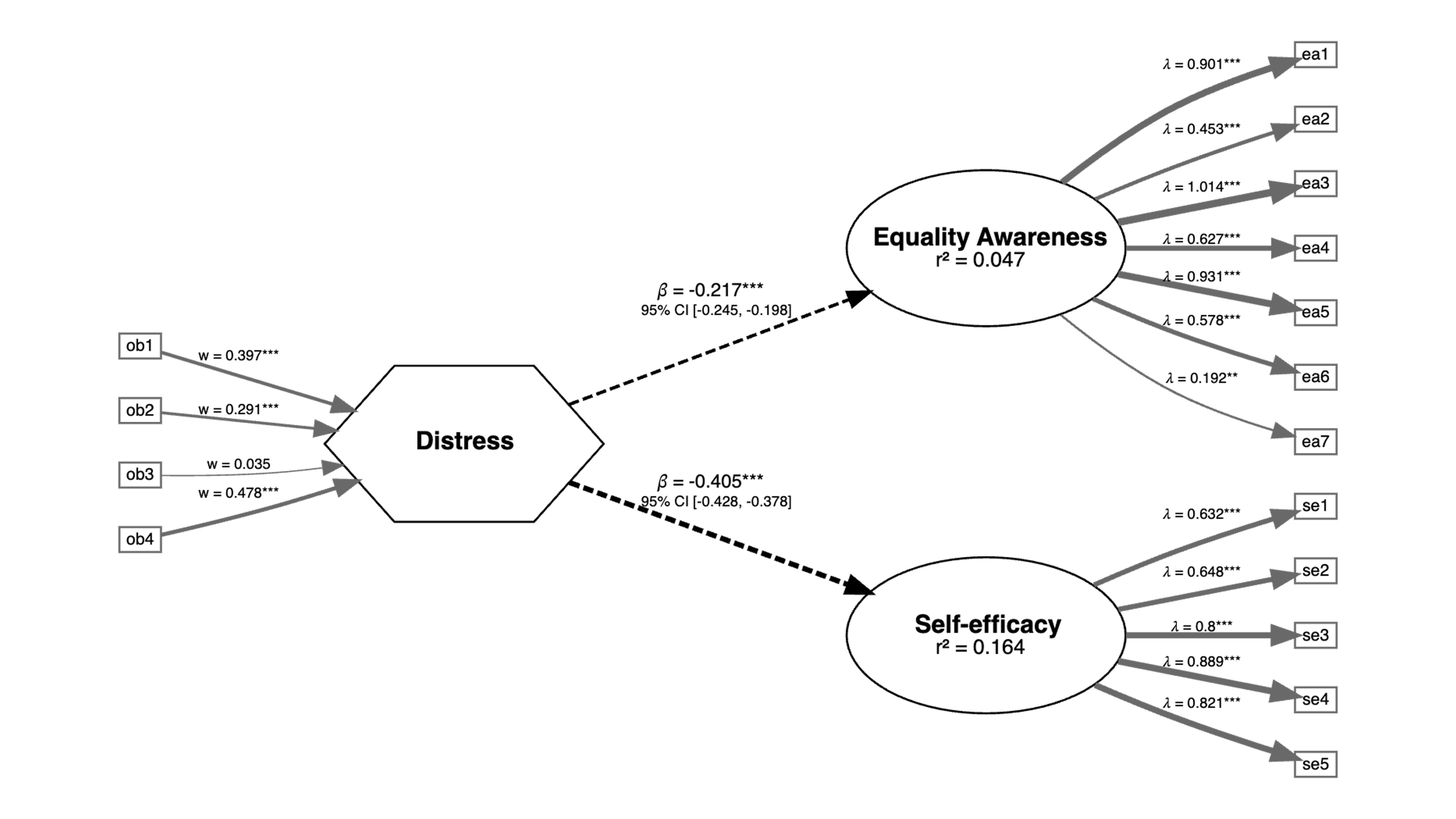

Supplement: S2 Fig — (TIF) [file pone.0326957.s004.tif]
