# Supplementary material for: Quantifying participant distress: Validity and applicability of a distress measure to evaluate harm in quantitative assessments
Source: PLoS One. 2025 Jul 2;20(7):e0326957. doi: 10.1371/journal.pone.0326957 (PMC12220986; doi:10.1371/journal.pone.0326957)
Supplement: S1 Table — A selection of existing measures to explore distress or similar aspects for research participants. (DOCX) [file pone.0326957.s005.docx]

**S1 Table. Similar distress measures**

A selection of existing measures to explore distress or similar aspects for research participants.

| Measure | Location | Focus | Data Collection Modality | Validation Procedures | Reference(s) |
| --- | --- | --- | --- | --- | --- |
| ﻿Reactions to Research Participation Questionnaire | ﻿Children’s Hospital of Philadelphia, Pennsylvania, USA | Clinical studies with children (n=96) and their parents (n=104) | 12-item self-facilitated questionnaire provided at the end of the interview. | - ﻿Cronbach’s alpha - ﻿Factor analysis | [11,12] |
| Shortened - Reactions to Research Participation Questionnaire | ﻿Seattle, Washington, USA | Study of women who experienced prior sexual and physical victimization (n=330) | 3-items at the end of a self-facilitated questionnaire focused on benefit, expected upset, and regret. | - Spearman correlations - Known groups testing | [8] |
| Self-reported ratings of a trauma assessment | St. Louis, Missouri, USA | Study of women who experienced prior traumatic assault or domestic violence (n=430) | 7-item self-facilitated questionnaire (paper or computer-based) provided at the end of the assessment. | - Known groups testing | ﻿[10] |
| Interview distress assessment | Australia | Study of army veterans with post-traumatic stress disorder (n=641) | 1-item asked at the end of enumerated interview. | - Known groups testing | [7] |
| ﻿Coercion assessment scale | Wilmington, Delaware, USA | Study of misdemeanor drug court clients (n=84) | 8-item self-facilitated questionnaire which included other questions after the informed consent discussion. | - ﻿Calculation of inter-item correlations and item-total correlations - Cronbach’s coefficient alpha - Exploratory factor analysis (EFA) - Discriminative validity (known-groups testing) | [9] |
| ﻿Quality of informed consent | Boston, Massachusetts, USA | Study of the consent process in adult cancer clinical trials (n=287) | 34-item self-facilitated questionnaire mailed to participants. | - Content and face validity - Test-retest reliability | [13] |
