# Supplementary material for: Quantifying participant distress: Validity and applicability of a distress measure to evaluate harm in quantitative assessments
Source: PLoS One. 2025 Jul 2;20(7):e0326957. doi: 10.1371/journal.pone.0326957 (PMC12220986; doi:10.1371/journal.pone.0326957)
Supplement: Attachment — Submitted filename: Responses to Reviewers.docx [file pone.0326957.s008.docx]

| **Section** | **Comment** | **Response** |
| --- | --- | --- |
| **Editor comments:** | | |
| Abstract | I miss the results and conclusion of the scale development and measurement properties in the abstract. | Updated the abstract to better summarize the validation results.  *“We present the measure’s conceptual and empirical development and examine of the validity of the measure through data from Cambodia and Nepal (n=4,674) using Partial Least Squares Structural Equation Monitoring (PLS-SEM) for formative measurement model assessment.*  *The measure is shown to have content and face validity; anticipated divergence with two reflective constructs; low collinearity; structural validity; and construct validity through known groups testing.”* |
| Introduction | Authors state “Ultimately, these considerations aim to mitigate potential distress and reduce the extractive nature of enumerated surveys” in the introduction section. How do the considerations above reduce the extractive nature of enumerated surveys? | Removed the second part of the sentence to remove the reference to extractive surveys. This makes the argument more clear. |
| Introduction | Authors state “Sample sizes are often larger than qualitative studies in order to ensure statistically significant analysis”. Rather than pointing to a significant analysis, adequate sample sizes are necessary to ensure statistical power for our study. Then our results can be generalized. Statistical significance is not directly determined by sample size alone. Please change "statistically significant analysis" to "statistical power and generalizability of results". | Updated as recommended. |
| Introduction | The third paragraph is unclear. Please explain how the sensitive topics mentioned can lead to distress reactions. | Clarified the paragraph to describe how discussing sensitive topics can lead to an emotional response which is described in literature as “distress”. |
| Introduction | The following statement, "However, there are limited tools to track and evaluate the comfort and ease - described in this study as distress - of participants in the enumerated surveys" is also unclear and needs to be referenced. What tools are available to assess the construct mentioned? Is the construct "comfort and ease" (of what?) or "distress" (of what?)? What type of participants (e.g., general population, adults, patients)? In the Background section, you can summarize (in 1 to 3 sentences) Table 1 in the text. | As recommended below, this paragraph has been merged with the background section and three justifications provided for this new measure. |
| Background | Please contextualize more quantitative studies. You only mention this term in your last paragraph, but the term is important throughout the manuscript. | Clarified the meaning of quantitative forms of research in the second paragraph |
| Background | Please consider presenting the Introduction and Background sections together. | Merged these sections as recommended. |
| Background | There are some scales as described by the authors. Why should a new scale be developed? | We have provided three more clear justifications for a new measure.   1. Focus (sociological research rather than medical) 2. Context (enumerated surveys in people’s homes rather than hospitals) 3. Geography (non-Global North) |
| Background | What do you mean by “field research in the Global South”? | Clarified this to be more specific to the geographic regions included (south and southeast Asia) as well as clarifying the focus on international development activities. |
| Methods | The subtitle “Approach” should be changed to “Methods”. | Updated as recommended. |
| Methods | Did you review the literature to gain insight into the development of the scale? Did you review the ISOPOR recommendations for instrument development | Updated to clarify the rapid design of the distress measure as necessitated by the ongoing process of piloting the WASH-GEM. Also clarified that for the full WASH-GEM tool we followed the procedures for social measure development as described here 10.3389/fpubh.2018.00149) |
| Methods | The methods section is unclear. Please be more specific: |  |
| Methods | 1 - How did the team identify the four aspects of potential distress? How was this assessment conducted? | This paragraph has been updated to more clearly explain the emergent process and the who specifically participated in the process. We have also added more clarity under the construct validity sub-heading in the measure validation section. |
| Methods | 2 - Were the team experts in the field? If so, how were they selected and how was their expertise assessed? |  |
| Methods | 3 - The following statement needs to be improved: "It should be noted that the Distress Scale, in a similar fashion to the Coercion Assessment Scale". What do you mean by "fashion"? What does the Coercion Assessment Scale assess? | This has been removed for clarity. |
| Methods | 4 - The “Programmatic context” paragraph is unnecessary because you already briefly stated it before. | Removed as recommended. |
| Methods | 5 - Details of the sampling methods (population, selection, etc.) used in the current study should be reported. | Added as recommended. |
| Methods | 6 - In Table 2, the first item seems to apply more to interviewers than to participants. The other items are more directed at the participants. Is this correct? The answer/scoring is not clear. For example, how will participants score “minimal” in ob2? Why are there different numbers (1/3, 2/3)? | Thank you for your comment. This item has to do with the rapport and tension between the enumerator and participant. The table and text have been updated to clarify this. |
| Methods | Please delete “by the first author” in the subtitle 3.4. | Removed as recommended. |
| Methods | The term “Item Validity” should be changed. This is not a measurement property (http://dx.doi.org/10.1016/j.jclinepi.2010.02.006). | Thank you for your comment. We have updated to item quality as per the recommendation of reviewer 2. |
| Methods | Did you assess the content validity of the scale with experts and participants? If so, how did you conduct the evaluation? | Yes, his was done with a series of expert reviews. We have now clarified this and described the content validation procedure in a labelled paragraph. We did not test content validity with participants as this is not aligned with the measure’s strategy, we instead validated with enumerators. |
| Methods | In your hypothesis test, poorer, older, and less educated people would have higher levels of distress than who? How high a level? Did you base your hypothesis on the literature or was it your experience? | We have clarified who we tested this against and also how the groups were identified. *“…that poorer, older, and less educated people would have higher levels of distress than their wealthier, younger and higher educated counterparts. The groups were identified through observations of the types of people who experienced distress during the rapid pilot and discussions with the program managers and enumerators. Each of these groups was tested separately for each item.”* |
| Methods | Is the Distress scale based on a reflective or formative model? If it is a formative model, structural validity and internal consistency should not be assessed. If it is based on a reflective model, you can perform factor analyses and add more details to the analysis (e.g., method estimation, rotation, etc.). | Thank you for this helpful comment. We have reframed the construct as formative and updated the methods as required. |
| Methods | Please change “Internal Reliability” to “Internal Consistency”, according to the recommendations (http://dx.doi.org/10.1016/j.jclinepi.2010.02.006). | Removed internal consistency as this is not required of formative measures. |
| Methods | What "key groups" did you use to assess measurement invariance? | Removed measure invariance procedures as this is not necessarily required of formative measures |
| Methods | Did you have missing data? If so, how did you deal with it? | This was hidden in the middle of a paragraph. We have moved it up to be in the datasets section. |
| Results | In the Results section, you should say "Construct validity through known groups" instead of "Content validity through known groups". Please use specific guidelines/checklists to report the Results section. | Updated as recommended. |
| Results | Did you adjust the model based on the modification indices of the factor analysis? This may improve the RMSEA. | Removed as no longer required. |
| Results | Why did you use a bi-factor model for scoring if the scale is unidimensional? | Removed as no longer required. |
| Discussion | “4.7 Limitations” should be moved to the end of the Discussion section. | Revised as recommended. |
| Discussion | Please revise your results and discussion based on my previous comments. | Revised as recommended. |
| General | I hope these comments are helpful. | We greatly appreciate your detailed and helpful comments. |
| **Reviewer #1:** | | |
| General | I found that the paper is well motivated. It developed a distress scale to evaluate distress in quantitative surveys. The authors proposed four aspects of potential distress and translated each of them into an item. In general, the paper is well written. I only have one concern about methodological aspect used. As stated by the authors, each of the four items represents one aspect of distress. Accordingly, the four items are formative indicators of distress. Using factor models (EFA/CFA – models for reflective indicators) for evaluating their properties is subject to misspecification and irrelevant item properties. The authors may refer to Hair et al. (2014) for assessing formative indicators. Hair JF, Hult GTM, Tingle C, Sarstedt M, (2014) A Primer on Partial Least Squares Structural Equation Modeling (PLS-SEM), Sage. | Thank you for this helpful feedback. We dug into this literature and now present the tool as a formative measure. |
| **Reviewer #2:** | | |
| Methodology and Technical | The authors conducted a study to develop a scale measuring distress caused by enumerated surveys. This is a necessary effort as discussed in the manuscript, and I appreciate the work put into the study. However, I have several methodological and technical concerns that may adversely impact the quality of the research: | Thank you for your helpful comments. The other reviewer and the editor recommended that we frame the tool as a formative rather than a reflexive construct, which reshapes the analysis direction away from a scale (reflective measure). We now use formative measure analysis procedures with PLS-SEM. |
| Item indices misinterpretation | On page 7, the reported item indices are about item quality rather than item validity. These indices do not address "the extent to which the items represent a range of experiences," as stated. In addition, there are different frameworks for evaluating item quality. Did the authors use Classical Test Theory (CTT) or Item Response Theory (IRT)? If IRT was used, please specify the model and report relevant software for your analysis. | Thank you for this clarification. As we are no longer presenting the measure as a scale, this is no longer required. Previously we had used CTT. |
| Construct validity | The study lacks an evaluation of convergent and divergent/discriminant validity, which are standard components of construct validation. Assessing group differences in distress by SES, age, and education level alone may be insufficient. In addition, if empirical studies exist that support these group differences, please discuss and cite them. | This is well noted. In alignment with the other reviewer’s comments, we now use formative construct validation procedures and explore convergent/ discriminant validity against other gender equality measures within the WASH-GEM. |
| Known-Groups Analysis | The role of known-groups analysis in informing content validity is unclear. Please clarify how this analysis supports the claim of content validity on page 11. | Thank you for this comment. We have clarified who we tested this against and also how the groups were identified. *“…that poorer, older, and less educated people would have higher levels of distress than their wealthier, younger and higher educated counterparts. The groups were identified through observations of the types of people who experienced distress during the rapid pilot and discussions with the program managers and enumerators. Each of these groups was tested separately for each item.”* |
| Number of items | The small number of items is concerning, and the justification that “there was not time or opportunity to identify a wide range of items and use traditional scale development procedures to drop items which did not fit in the model” is problematic. | This is well noted and led to our framing of the tool as a formative rather than a reflexive construct. |

**Journal requirements:**

When submitting your revision, we need you to address these additional requirements.

1. **Updated.** Please ensure that your manuscript meets PLOS ONE's style requirements, including those for file naming. The PLOS ONE style templates can be found at
   [https://journals.plos.org/plosone/s/file?id=wjVg/PLOSOne_formatting_sample_main_body.pdf](https://url.au.m.mimecastprotect.com/s/6pQqC2xM9AUpNX0POT9tAH5Cv_K?domain=journals.plos.org) and 
   [https://journals.plos.org/plosone/s/file?id=ba62/PLOSOne_formatting_sample_title_authors_affiliations.pdf](https://url.au.m.mimecastprotect.com/s/02n8C3QN90Sp5lW60TvurHQS2Hl?domain=journals.plos.org)
2. **Updated.** Please include a complete copy of PLOS’ questionnaire on inclusivity in global research in your revised manuscript. Our policy for research in this area aims to improve transparency in the reporting of research performed outside of researchers’ own country or community. The policy applies to researchers who have travelled to a different country to conduct research, research with Indigenous populations or their lands, and research on cultural artefacts. The questionnaire can also be requested at the journal’s discretion for any other submissions, even if these conditions are not met.  Please find more information on the policy and a link to download a blank copy of the questionnaire here: [https://journals.plos.org/plosone/s/best-practices-in-research-reporting](https://url.au.m.mimecastprotect.com/s/JUSoC4QO9DSBpORM3hjC1H46vE5?domain=journals.plos.org). Please upload a completed version of your questionnaire as Supporting Information when you resubmit your manuscript.
3. **Updated**. Thank you for stating in your Funding Statement:
   This work was supported by the Australian Government’s Water for Women Fund [Grant number WRA-034 - JW]. The funders did not play any role in the study. Please provide an amended statement that declares *all* the funding or sources of support (whether external or internal to your organization) received during this study, as detailed online in our guide for authors at [http://journals.plos.org/plosone/s/submit-now](https://url.au.m.mimecastprotect.com/s/XDvVC5QPWESZV7w54tlFxHkBgOD?domain=journals.plos.org).  Please also include the statement “There was no additional external funding received for this study.” in your updated Funding Statement. 
   Please include your amended Funding Statement within your cover letter. We will change the online submission form on your behalf.
4. **Updated.** Your ethics statement should only appear in the Methods section of your manuscript. If your ethics statement is written in any section besides the Methods, please move it to the Methods section and delete it from any other section. Please ensure that your ethics statement is included in your manuscript, as the ethics statement entered into the online submission form will not be published alongside your manuscript.
5. **Now published and updated.** We note that you have referenced "MacArthur J, Basnet MP, Budhathoki R, et al." and "MacArthur J, Chase RP, Gonzalez D, et al." which has currently not yet been accepted for publication. Please remove this from your References and amend this to state in the body of your manuscript: (MacArthur J, Basnet MP, Budhathoki R, et al." and "MacArthur J, Chase RP, Gonzalez D, et al." [in preparation) as detailed online in our guide for authors
   [http://journals.plos.org/plosone/s/submission-guidelines#loc-reference-style](https://url.au.m.mimecastprotect.com/s/gEsDC6XQ07trnvZj8I9H2H5V-6B?domain=journals.plos.org).
6. **Updated.** Please remove your figures from within your manuscript file, leaving only the individual TIFF/EPS image files, uploaded separately. These will be automatically included in the reviewers’ PDF.
